# Perceptions, cartographies, and ‘cartographies of time’ in the political novel

**DOI:** 10.12688/openreseurope.19924.1

**Published:** 2025-05-19

**Authors:** Tomasz Mizerkiewicz

**Affiliations:** 1 Adam Mickiewicz University in Poznan Faculty of Polish and Classical Philology, Poznań, Greater Poland Voivodeship, Poland

**Keywords:** perception, cartography, political novel, Milan Kundera, Annie Ernaux

## Abstract

The paper examines the relation of the form of political novel to perceptions. Three possibilities are analysed. First, when the political novel shares perceptions of the hegemonic power. Second, when the novel trains how to suspiciously and critically reveal hegemonic manipulations of perceptions. Third, where the form of the novel enhances and emancipates perceptive faculties of readers and allows them to project new democratic activities. This three-fold relation is also referred to cartographic activities (including cartographies of time) as depicted in political novels.

## Plain language summary

The article describes how those in power influence citizens’ perceptions of space and time, and shows how political novels can support the ‘eye in the sky’ perspective of the regime, or criticise and undermine it. The most interesting occurs to be the third option, when political novels invent space and time for the new and free democratic activities.

Milan Kundera’s novel *The Book of Laughter and Forgetting* (1981) was written in 1978, shortly after Kundera had decided to emigrate from communist Czechoslovakia because he was persecuted by the regime as a dissident for his engagement in the democratic processes of the ‘Prague Spring’ in 1968. ^1^ The novel describes the various consequences of the disappointing political enterprise of an entire generation of left-wing intellectuals. Their starting point was the installment of the communist regime, which they had warmly supported in their youth:

In February 1948, Communist leader Klement Gottwald stepped out on the balcony of a Baroque palace in Prague to address the hundreds of thousands of his fellow citizens packed into Old Town Square. It was a crucial moment in Czech history – a fateful moment of the kind that occurs once or twice in a millennium.
Gottwald was flanked by his comrades, with Clementis standing next to him. There were snow flurries, it was cold, and Gottwald was bareheaded. The solicitous Clementis took off his own fur cap and set it on Gottwald’s head.
The Party propaganda section put out hundreds of thousands of copies of a photograph of that balcony with Gottwald, a fur cap on his head and comrades at his side, speaking to the nation. On that balcony the history of Communist Czechoslovakia was born. Every child knew the photograph from posters, schoolbooks, and museums.
Four years later Clementis was charged with treason and hanged. The propaganda section immediately airbrushed him out of history and, obviously, out of all the photographs as well. Ever since, Gottwald has stood on that balcony alone. Where Clementis once stood, there is only bare palace wall. All that remains of Clementis is the cap on Gottwald’s head ( 1 on page 10).

The inaugurating passage from Kundera’s novel includes a reference to the imprinted perception of a powerful political event. The image of the place and the moment when the Czech and Slovak communists seized power is reproduced, i.e. petrified as a founding perception, an iconic image of the new order. The reader of the novel is aware of the fact that this perception – among many others – was intended by the powers to control the perceptual activities of the citizens and to steer their emotions. This is what every power does, and we can use the political novels of socialist realism as an example of how they are in sync with the processes of stabilising and reproducing this mode of perception of the dominant political order.

The passage quickly complicates this pathetic style of reproducing the founding perception, as the narrator tells us a dramatic story of manipulations concerning the famous primordial scene. The novel seems to belong to the critical narratives that expose these cynical operations on the perceptual field of politics. The erasure of the cursed politician from iconic images has analogous practises in the political novels of socialist realism, where the new editions of books written by the servants of the regime were given a discreet makeover or editing in order to adapt the book to the latest demands of the party. This is why every important political novel of socialist realism was by definition a new and different edition, although these acts of their political design were as secret as the removal of Clementis from his picture with Gottwald. Exposing this kind of manipulation is the domain of the critical perception of political novels, which devote most of the reader’s attention to directing his or her suspicions towards the official field of perception of the political order. The tragic and somewhat comical and stubborn persistence of Clementis – his fur hat on Gottwald’s head – was a training in mistrust and critical analysis of every official use of perceptions in the public sphere by the hegemonic powers of the time. The development of mistrust as the basic activity of some political novels is necessary to break ties with the most aggressive perceptive imprints imposed on citizens by rulers.

Right after the initial part of the book, the narrator follows the Czech resistance hero of the Prague Spring, who is spied on by the secret service but nevertheless decides to take a risky journey to his former lover. Kundera does not fully advocate this independent activist, as the man wants to destroy some letters in which he tells the unattractive girl from his past of his true love. Just like the communist regime, he is deeply driven by the urge to manipulate the perception of the past. The novel definitely does not limit its perceptual adventure to the model of the suspicious vivisection of official political perception. Chapter by chapter, it builds up a composition that has been called “a novel in the form of variations” ( 1 139), in which the same scenes and problems reappear in unexpected places and sometimes in a new guise. The iconic scene on the balcony is described a few more times, as are the themes of memory, manipulation, disappointment and other issues raised in the opening paragraph. The form of the novel aims to achieve maximum concentration on the chosen themes. This is why the author has accepted to call his book “phenomenological” ( 8 on page 35), with some reservations about the important difference between the philosophical and the artistic treatment of phenomenological enterprises. The novel aims to become a unique tool for long-term study, in which the composition and repetition of perceptions should lead to an awareness of them, increase the intensity of the phenomena studied and maximise concentration on them. The novel strengthens the reader’s perceptive capacity and prepares him or her for independent activity, including in the political sphere.

This allows us to recognise the threefold or three-dimensional relationship of political novels to the perceptions shared in public and private life. Political novels can be involved in the reproduction of the perceptions disseminated by the hegemonic powers or by the ‘eye in the sky’ that controls the entire field of sensibility with this pattern of perception. Political novels can then draw our attention to the possibility of critically analysing these perceptions and train our suspicious minds to detect manipulative practises. Finally, the most intriguing political novels decide to take one step further. They turn into machineries in which some perceptual skills are developed through the novel itself, confronting both the ‘royal’ perceptions of the hegemonic powers and the suspicious perceptions of the critical dissidents. The novelistic perceptual play of the motifs, their repeated appearance and their variations are intended to emancipate the reader’s perceptual capacity to a certain degree, to enable them to act as free political actors and to create new spaces for their possible communities. The novel as a form of liberation of perceptual capacities and the intensification of these perceptual capacities becomes a means of rethinking and co-creating these new spaces. This becomes particularly clear when we think of the book written by the Czech dissident who was forced to leave his country after the failed democratic revolution.

To make some further comments on the way in which the political novels can help to recreate spaces for new possible communities, we need to consider the spatial and cartographic manoeuvres undertaken in this particular genre. It is easy to foresee that these manoeuvres would also bring the political novel into a triple or threefold relationship with political practises.

First, they analyse (or, in worse cases, participate in) the process of conquest and possession of a territory in geographical, sociological and other terms. Cartography is one of the tools used by the hegemonic power to establish itself in a country and control the occupied territory, and has therefore often been criticised. The iconic image conveys the desire to take possession of the centre of the Czechoslovak capital and to gain solid control over the entire territory of the country in order to stand in the ‘eye-in-the sky’-position of a panoptic controller. The cartographic coverage of the entire country is typical of the imaginary of political novels supporting the regimes.

Second, in some cases the political novel participates in or notices acts of resistance and subversion of the dominant cartographic practises of hegemonic powers. There, cartography is treated as purely constructed, artificial, or at best the result of someone else’s interpretation. These political novels trace possibilities of deterritorialisation – to use Félix Guattari and Gilles Deleuze’s term (see 9) – as in the case of *The Book of Laughter and Forgetting*, the misleading journey of the dissident who escapes the control of the spying secret service agents. Any attempt by cartography to fully map the country and extend the control of power over the citizens is in such cases undermined or mocked causing power to lose its solid grounding. The counter-cartographic intention is a dominant aim of this political novel. Its spatial cartographic representation is inherently interpretative, narrative and constructivist – it is the result of the discursive illusion and the performative practises of rhetoric. To fully achieve the critical intention, this idea of cartography resembles the famous story by Jorge Luis Borges, in which the map is indistinguishable from the covered land itself. One cannot distinguish what is a ‘real’ map and what is a real land, any act of grounding power hangs in a vacuum.

 Thirdly, the political novel has a potential – albeit not always utilised – to be part of practises that go beyond this opposition and seek a sphere that Alain Badiou would call the “subtraction” (see 10) of the revealed opposition. In this third activity, the political novel is an agent that creates effective and useful political cartographies, which seems necessary if one wants to plan and carry out an intervention. Its ontology would be the greatest challenge, because we must understand the political novel as using, in some situational contexts, invented and different models (as defined by Alva Noë, see 11), miniatures, copies, substitutes, graphs, dummies, pattern volumes and other forms of representation and cartography that are part of some practises. Here the constant play between materialisation and dematerialisation of the represented continues. The political novels seem to be embedded in numerous everyday activities where material models are constantly produced and used as constellations of objects when we explain how to get somewhere, or as schematic pictures we draw for a random user to help them find their way around a place. This also means that in order to uncover this kind of political cartographic activity of the novel, we also need to change the way we reflect on literary forms.

In his new reading of Bruno Schulz, the Polish critic Paweł Tomczok pays attention to the fact that the story shows a miniature of the city described by the narrator. This prompts him to look at the ontology of the narrator of the story itself (see 12). Similar to the play of materialisation and dematerialisation of the worlds depicted in the model called miniature, the narrator is also treated as involved in the constant drama of his presence/absence. There are moments when the narrator is only an abstract dimension of the text, as most contemporary theories would have us believe, but in other cases he obviously shows his bodily gestures, comments on the timbre of his voice and behaves like a narrator from the oral tradition of literature. The story is thus to be read as a sequence of materialisations and dematerialisations of the narrator. If the narrator and other novelistic instances were treated in this way – that is, in a way that comes close to what has already been called ‘postcritical literary studies’ – then the novel dealing with political issues, could be usefully studied as a valuable practise of political cartography.

In the case of Kundera’s novel, we should consider the status of its metanarrative parts. They not only undermine the illusion of the reality of the political world depicted, or reveal its fictional features, but also introduce a kind of writer’s workshop in which the novelistic reality is being modelled. Kundera’s narrator tells us how and why he creates some characters, where he is and what knowledge he already has about the composition of the novel, including his intentions regarding the characters and his hesitations or feelings. Here, too, we follow a constant drama of the narrator’s presence and absence, his materialisation and dematerialisation. Another example is the material archives presented in the book. Some of the characters are in search of the lost letters that contain a precious part of their past. The same could be said about the photographs, such as the initial picture of Gottwald and Clementis. In a story about students trying to understand the play *Rhinoceros* by Eugène Ionesco, there is an important scene in which the students perform part of the drama unsuccessfully. In all these cases, we can observe practises of dealing with the material carriers of some representations of the world.

Why should we trace this balancing of the political novels in the material and immaterial characteristics of cartographic images? I would answer that in politics you are always – to a certain extent – dealing with substances, materials, countries, bodies or properties. This field cannot be limited or reduced to its discursive or linguistic representations. For this reason, some of the political novels are involved in processes of materialisation and dematerialisation, where cartography is also understood as a broad field of everyday practises of schematic outlining, situational drawings, the creation of miniatures and simplified models. We always need some cartographic tools and we always produce them in order to intervene effectively in the world around us, in its discursive and material qualities. The cartographies of political novels are usually aware of their abstract and constructed character and they still pay attention to violating and oppressive influences of the ‘hard’ or solid cartography of the dominant powers. But they do not cease to continue their own cartographic endeavours as they create many new “graphs, maps, trees” (to use the title of Franco Moretti’s monograph, see 13) to operate with given symbolic and material substances and experimentally map some new spaces for political activity. Kundera’s novels are always about the search for a balance between the lightness and heaviness of being. His novels introduce us to the tension between these two unavoidable ontologies of political cartography and prepare us to navigate it. To be a conscious and active agent in this process or tension, the novel provides us with a special perceptual training, helps us to maximise our focus on these phenomena and teaches us how to study and use them in our possible future activities.

There is, however, at least one other aspect of this threefold involvement of political novels in spatial and cartographic action, which has become increasingly apparent in recent times, when studies of the new temporalities in literature are developing rapidly. The passage from Kundera’s book had strongly emphasised the importance of temporal experience in relation to political events. We could call it, using Daniel Rosenberg and Anthony Grafton’s term, as the “cartography of time” (see 14), where the political implications of spatial activities are fused with the political implications of temporal activities.

This allows me to move on to my second example, the book by the French writer Annie Ernaux, *Les années* ( *The Years*, 2017). I do not think about this case without hesitation, because her autobiographical prose is more informal and less like the classic novel compared to Kundera’s book. Nevertheless, the main pattern of the narrative is borrowed from the canonical novel *Une Vie* ( *A Life*) by Guy de Maupassant, in which the life of a single woman is presented in a grand political and historical panorama. However, it is too tempting for me not to comment on this example, because the writer’s entry into the political sphere of her time is a series of idiosyncratic perceptions, intensities of past images, intensities of past voices, intensities of past words, songs, etc. The book begins and ends with an enumeration of the loose and disorganised representations of some unforgettable perceptions and sensations of materialities. Let me now explain why these perceptual streams of private and public life events, analogous to Kundera’s composition, strengthen the reader’s perceptual capacity. It would be easy to find paragraphs in which Ernaux lets the perceptual impressions of the regime of Charles de Gaul or Nicolas Sarkozy sink into the narrative. The same could be said of her involvement in the feminist resistance and revolts, some of which were very successful and effectively and critically subverted the regime’s perceptions. The same could be said of Ernaux’s attitude to cartography. She has perfectly depicted the French cartographic hegemonic obsession revealed during the Algerian War, when maps were used as tools of the dominating colonial power. We can also see how the female subject in the male cultural system forced her to look for some deterritorialisations with which she could completely subvert the patriarchal mapping of social orders. This eventually led her to write the story in which she created her own cartographies using the material carriers such as photographs, family films and many other common and tangible models. Apart from this, her book was also intended to develop the cartographic affordances of the political novel in some selected temporal images. This interference of spatial and temporal substances and symbolisations could be called a novelistic ‘cartography of time’, which we may recognise in the following paragraphs:

If we tried to enumerate the things that happened outside us, after September 11 we saw a rash of swift-moving events, a series of expectations and fears, interminable times and explosions that paralyzed or deeply distressed us – “nothing will be as it was before” was the dominant theme – and then disappeared, forgotten, unresolved, and commemorated a year or even a month later, as if they were ancient history. There was April 21, the war in Iraq, which fortunately did not include us, and the death of John Paul II, another pope whose name we hadn’t retained, let alone his number, the bombing of the Atocha station, the great festive evening for the No vote in the European Constitution referendum, the incendiary nights of rioting in the banlieue, Florence Aubenas, the London terrorist attacks, the Lebanon War between Israel and Hezbollah, the Indian Ocean tsunami, Saddam rooted out of a hole and hanged (no one knew when), nebulous epidemics, SARS, avian flu, the chikungunya virus […]. Everything seemed overwhelming. The United States was the master of time and space, which it occupied according to its needs and interests. Everywhere the rich grew richer and the poor grew poorer. People slept in tents all along the Boulevard Périphérique. The young sneered “Welcome to a world of shit” and briefly rebelled. Only the retired were satisfied, and sought advice on how to save and spend their money, traveled to Thailand, shopped on eBay, and visited Meetic online dating. Where could revolt come from? ( 15 on page 142–43)
What matters to her, on the contrary, is to seize this time that comprises her life on Earth at a given period, the time that has coursed through her, the world she has recorded merely by living. She has mined her intuition of what her book’s form will be from another sensation, the one that engulfs her when, starting with a frozen memory-image of herself with other kids on a hospital bed after tonsil surgery, after the war, or crossing Paris on a bus in July of ’68, she seems to melt into an indistinct whole whose parts she manages to pull free, one at a time, through an effort of critical consciousness: elements of herself, customs, gestures, words, etc. The tiny moment of the past grows and opens onto a horizon, at once mobile and uniform in tone, of one or several years. Then, in a state of profound, almost dazzling satisfaction, she finds something that the image from personal memory doesn’t give her on its own: a kind of vast collective sensation that takes her consciousness, her entire being, into itself. She has the same feeling, alone in the car on the highway, of being taken into the indefinable whole of the world of now, from the closest to the most remote of things.
So her book’s form can only emerge from her complete immersion in the images from her memory in order to identify, with relative certainty, the specific signs of the times, the years to which the images belong, gradually linking them to others; to try to hear the words people spoke, what they said about events and things, skim it off the mass of floating speech, that hubbub that tirelessly ferries the wordings and rewordings of what we are and what we must be, think, believe, fear, and hope. All that the world has impressed upon her and her contemporaries she will use to reconstitute a common time, the one that made its way through the years of the distant past and glided all the way to the present. By retrieving the memory of collective memory in an individual memory, she will capture the lived dimension of History. ( 15 on page 150–51)

This longer quotation once again opens up the threefold relationship of the political novel to temporal questions. We have an example of a dominant power, the United States, dominating time and space globally in the first decade of the new millennium. The book also refers to some other masters of time, namely economic and marketing strategies that synchronise temporal rhythms on a global scale. The helplessness of this situation, in which no revolt is possible, leads to a bitter and critical counter-narrative of the radically asynchronous and chaotic mass of events. This kind of narrative prevents, to a certain extent, the influences of the global cartography of time. It exposes its totalising, homogeneous, hurtful and constructed ontology. And this is where Ernaux decides to go one step further. In all these years, she could not begin her story about herself as a woman in time because she had no basic perception or reason that could be distinguished from this overwhelming mass of events and their imposed perceptions. Instead of reproducing the media and political images and instead of merely melancholically subverting them, she had to transform her book, as we could read, into a machinery that maximised her idiosyncratic sensations of peculiar personal images of time, studying their intensity and thus liberating her perceptive faculties. The moment of immersion in the frozen images of the past was associated with the sensation of melting the materiality of these images, loosely dissolving them and recovering the consciousness of this time, of sharing all substances with others. This is all the more radical when we consider how worthless these materialities of time can be decades later, when they are no longer a commodity for others.

In order to achieve this kind of temporal liberation of the past, Ernaux must perceive it as a material mass of impulses. This is why she even uses the metaphor of ‘tone’ and we could associate her writing to the mood or atmosphere in a literary work, something that Hans Ulrich Gumbrecht has taken up again for literary studies by emphasising its importance for the dimension of presence (see 16). This is exactly what seems to happen in the quoted paragraphs, the liberating potential of the idiosyncratic perception of the past event is tied to its fleeting presence, or more precisely: To the immersion in its increasingly felt interplay of presence and absence (latency, as Gumbrecht would put it). The main aim of Ernaux’s narrative is “to reconstitute a common time” and this time is common if it has some material properties that can be derived from the image. The time is common because each substance was basically common in a certain time, which is even more vivid after decades in which they have usually lost their significance for the political cartographies of the ‘masters’. And this strange ontological-temporal materiality opens up the possibility of drawing trajectories of common time, in which we associate spatial and temporal activities with substances, properties or bodies. Ernaux presents her own trajectory, but also invites us to read other political novels as other trajectories, all of which together develop cartographies of time formed by the activists of common time.

In conclusion, I would like to emphasise once again the gradual involvement of the political novels in questions of perception. The main reason for this involvement is the tendency of every power to control the entire field of perception, be it political, economic, social, religious or otherwise. The political novels usually portray this tendency, but in the most servant cases they also help the regime to reproduce itself by hurting the lives of the citizens in perception. This harmful influence leads many political novels to elaborate a critical and suspicious analysis of these ‘royal’ perceptions, training readers to expose the perceptual manipulations of those in power. The most productive seem to be the political novels that add another aspect to this opposition, and our analysis should be ready to support them in this ambition. They turn into enhancements of our perceptual capacities and rely on our growing perceptual skills in their aim to pave the way for an independent activist cartography and a cartography of time for possible political action. To achieve this goal, they need to change our usual way of dealing with literary texts. They are not purely discursive and linguistically abstract forms, but also engage with the interplay of the linguistic and materialistic features of composition. In this way, political novels can intervene in the cartographic field and become more and more aware of how they can effectively combine the constant drama of their discursive and material properties to design new cartographies and new cartographies of time for successful future democratic activities.

## Ethics and consent

Ethical approval and consent were not required.

## Data Availability

No data associated with this article.
