# Supplementary material for: Perceptions, cartographies, and ‘cartographies of time’ in the political novel
Source: Open Res Eur. 2025 May 19;5:137. [Version 1] doi: 10.12688/openreseurope.19924.1 (PMC12308170; doi:10.12688/openreseurope.19924.1)
Supplement: Peer review history

# Reviewer report: Reviewer response for version 1

**DOI:** 10.21956/openreseurope.21552.r54617

**Published:** 2025-07-29

**Authors:** Laura Lo Presti

**Affiliations:** 1 Università di Padova, Padova, Italy

**Perceptual Machines and Literary Cartographies**

**Summary**

 Tomasz Mizerkiewicz’s article proposes a stimulating and ambitious itinerary: to explore the political novel not only as a literary artefact, but as a perceptual and cartographic dispositif—a machine capable of replicating, challenging, or liberating political perception. The essay aims to reposition the political novel within a “threefold” matrix of perceptual relations: novels that reproduce hegemonic visions; novels that critique and deconstruct them; and, finally, novels that intensify perception and generate emancipatory political possibilities. By drawing a parallel with spatial and temporal cartographies—especially in the works of Kundera and Ernaux—the paper enters a fertile but dangerous interdisciplinary zone. As this review will argue, the cartographic conceptualization remains largely metaphorical, and would benefit from a deeper engagement with contemporary cartographic humanities. Through closer attention to authors like Ernaux and Kundera, and their treatment of memory, time, and perception, the article could more effectively position the political novel as an insurgent cartographic form—one that generates new perceptual and political geographies rather than merely representing existing ones.

 For a humanist map scholar, in a time of geopolitical cartographic overexposure, the proposal to explore the political novel not only as a literary artefact but as a perceptual and cartographic dispositif—a machine capable of replicating, challenging, or liberating political perception—is certainly timely and ambitious.

 The essay, in particular, aims to reposition the political novel within a “threefold” matrix of perceptual relations: novels that reproduce hegemonic visions; novels that critique and deconstruct them; and finally—and most compellingly—novels that function as perceptual laboratories, activating emancipatory faculties in the reader. By drawing a parallel with spatial and temporal cartographies—especially in the works of Kundera and Ernaux—the paper enters a fertile interdisciplinary zone. Yet, as this review will argue, the cartographic conceptualization remains largely metaphorical and would benefit from a deeper engagement with post-representational cartography (Dodge et al., 2009) and contemporary cartographic humanities (Rossetto and Lo Presti, 2024).

 My emphasis on mapping is not meant to be pretentious, but the title obsessively signals an alignment with the “cartographic turn” in the humanities, and the essay gestures toward the analytical promise of concepts like “cartographies of time” and “counter-mapping.” However, these concepts remain only partially sketched and are used in a rather superficial way. Despite references to Franco Moretti, Deleuze and Guattari, Badiou, and Grafton & Rosenberg (who are not geographers nor cartographers), the framework lacks engagement with a growing body of literature that directly theorizes the map-text nexus—and more.

 In sum, while the intuition is promising, the articulation remains tentative. The conceptual movement between literature and mapping is evoked rather than constructed. And this matters, especially when claiming to chart a new critical itinerary between narrative and power.

 To read the novel as a map is not new. Yet this piece seems unaware of the sedimented scholarship that would allow it to stand on firmer ground. Cultural geographers, literary theorists, and post-representational cartographers have already traced, layered, and counter-mapped this terrain (and it is surprising not to see any mention of their work).

 The article does not reference key figures in critical cartography, who have long interrogated the ideological and technical regimes embedded in map-making. Where are the epistemological breaks initiated by Brian Harley’s critique of cartographic objectivity? Or Matthew Edney’s historical unpacking of the “ideals” of map-making? Or Akerman’s work on colonial and postcolonial cartography? Or even Herb’s research on the cartographic construction of Germany and Eastern Europe? Without this background, it is difficult to distinguish between what is structurally cartographic and what is simply spatial or perceptual in literary form. Terms like “deterritorialization,” “counter-cartography,” or “new possible communities” are deployed without analytical precision. The transitions between metaphors (from map to miniature, archive to diagram) are too rapid, and the application to literary texts remains too abstract. The author’s reference to Borges’s tale of the map as territory is evocative but could have been strengthened by a dialogue with post-representational cartography, where maps are treated not as (false) images of reality, but as performative tools of world-making.

 Without a deliberate engagement with these traditions, the proposed bridge between novelistic form and cartographic modes remains suspended—conceptually adrift.

 More broadly, the article’s claim to originality would benefit from the vocabulary and methodological openness developed in the field of counter-mapping—especially where map and narrative intersect as spaces of resistance or care, capable of representing lived, embodied, and affective geographies of empowerment and endurance. Counter-mapping, as practiced by Indigenous groups, activist cartographers, and feminist geographers, is not just subversion; it is a reappropriation of mapping techniques to produce alternative spatial knowledges. In this sense, Ernaux’s reclaiming of time through memory fragments could have been framed as a **chronotopic **counter-map—a form of temporal insurgency against the linear histories imposed by state and media.

 Ernaux gestures toward a cartography of time, but the author does not engage with chrono-cartographic practices or the broader discussion on temporal geographies (e.g., Tally 2019; Lammes et al., 2018). The palimpsestic timescapes of Ernaux deserve closer attention—not merely as narrative devices, but precisely because they are modes of resisting linear historicity and nation-bound chronologies. A dialogue with thinkers such as Barbara Adam or Reinhart Koselleck could help flesh out this dimension.

 When reading the brief quotation from Ernaux, I could begin to see how it might be explained as a kind of perceptual cartogenesis—or even a micropolitical cartography of memory.

 Here the paper begins to speak the language of contemporary cartographic humanities, where maps are not only spatial diagrams but expressive practices, performances, and forms entangled with affect, memory, and subjectivity.

 How, for instance, does Ernaux’s fragmentary style affect perception—and even construct fragmentary maps? What does it mean, for instance, for Ernaux to “map” time? How is this mapping different from the techniques used by state chronologies or historical archives? Without this differentiation, the cartographic analogy remains static and uncritical. How does Kundera’s ironic detachment map a form of perceptual critique? Without close readings, the theoretical ambition floats in abstraction.

 To reposition the article within a fertile interdisciplinary constellation, one could reframe its key thesis as follows: political novels do not merely represent power—they territorialize it, subvert it, or unmap it. They operate like maps: delimiting, orienting, distorting, or opening new perceptual affordances. Some novels act like topographic instruments of control, others like cartographies of suspicion, and the most radical as insurgent maps: open-ended, unstable, affectively charged.

 I believe that we need to abandon the idea of the novel as a mirror and embrace it as a cartographic machine: not mimetic, but generative. The reader is invited not to receive the world, but to re-world it. This gesture, if expanded, could resonate with current debates in post-representational cartography (see Dodge, Kitchin and Perkins, 2009), which now finds a significant place within the cartographic humanities (Rossetto and Lo Presti, 2024).

 The opportunity here is not only to re-read novels through the lens of cartography, but to perform a fresh, updated mapping critique of the literary, where the act of reading itself becomes an act of situated mapping—sensory, political, and always partial.

 Mizerkiewicz’s article offers a provocative framework for rethinking political novels as cartographic agents of perception. Its strongest contributions lie in its focus on perceptual emancipation and the novel as a training device for political subjectivity. Yet to achieve the conceptual clarity and originality it aspires to, the paper must revise its cartographic lexicon, draw more extensively on the literature of critical mapping, and better integrate theory with close reading.

 To do so would not only strengthen its scholarly rigor, but also allow it to genuinely “co-create new spaces” where literary analysis, geography, and politics intersect—something deeply resonant with the ongoing project of the cartographic humanities.

If any, are all the source data and materials underlying the results available?

Yes

Is the work original in terms of material and argument?

Yes

Is the work clearly and cogently presented?

Partly

Is the argument persuasive and supported by evidence?

Partly

Does the research article contribute to the cultural, historical, social understanding of the field?

Partly

Does it sufficiently engage with relevant methodologies and secondary literature on the topic?

No

Reviewer Expertise:

I am a cultural geographer, expert in cultural mapping, cartographic humanities, critical cartography and their use in art, literature, media (and humanities more broadly).

I confirm that I have read this submission and believe that I have an appropriate level of expertise to confirm that it is of an acceptable scientific standard, however I have significant reservations, as outlined above.

## References

[1] : DECONSTRUCTING THE MAP. *Cartographica* .1989;26(2) : 10.3138/E635-7827-1757-9T53 1-20 10.3138/E635-7827-1757-9T53

[2] : Theorizing maps with literature. *Progress in Human Geography* .2014;38(4) : 10.1177/0309132513510587 513-530 10.1177/0309132513510587

[3] : The Routledge Handbook of Cartographic Humanities.2024; 10.4324/9781003327578 10.4324/9781003327578

[4] : Under the Map of Germany.2002; 10.4324/9780203433720 10.4324/9780203433720

[5] Rethinking Maps.2011; 10.4324/9780203876848 10.4324/9780203876848

[6] Time for mapping.2018; 10.7765/9781526122520 10.7765/9781526122520

[7] : James R. Akerman, ed. Decolonizing the Map: Cartography from Colony to Nation. *New Global Studies* .2019;13(2) : 10.1515/ngs-2019-4019 260-263 10.1515/ngs-2019-4019

[8] : Topophrenia.2019; 10.2307/j.ctv7r40df 10.2307/j.ctv7r40df

[9] : Time in Maps.2020; 10.7208/chicago/9780226718620.001.0001 10.7208/chicago/9780226718620.001.0001

[10] : Cartography: The Ideal and Its History, Chicago. *https://www.degruyterbrill.com/document/doi/10.7208/9780226605715/html* .2019; 10.7208/9780226605715 10.7208/9780226605715

[11] : Cartographies of Fictional Worlds. *https://www.tandfonline.com/doi/full/10.1179/174327711X13190991350051* .2011; 10.1179/174327711X13190991350051 10.1179/174327711X13190991350051

# Reviewer report: Reviewer response for version 1

**DOI:** 10.21956/openreseurope.21552.r55094

**Published:** 2025-06-23

**Authors:** Alina Bako

**Affiliations:** 1 Lucian Blaga University of Sibiu, Sibiu, Romania

This article aims for an ambitious goal, promising to reveal cartographies that are, however, only sketched out. Although the chosen corpus is highly rich, consisting of Milan Kundera's and Annie Ernaux's books, the promise is not fully fulfilled. Current working tools are discussed, which could lead to an understanding of literature from an innovative perspective. Nevertheless, the article uses fragments of concepts, such as those from Moretti's seminal study or the phrase “the cartographies of time,” which could have been related to the political chronotop, without justifying their presence or the role that the political novel might hold in a potential diagrammatic scheme, of a genealogical type. 

 Passages such as “The novel aims to become a unique tool for long-term study, in which the composition and repetition of perceptions should lead to an awareness of them, increase the intensity of the phenomena studied and maximise concentration on them. The novel strengthens the reader’s perceptive capacity and prepares him or her for independent activity, including in the political sphere.” contain redundancies and do not clarify the meaning that the article initially promised. We do not understand how a novel would become a tool for long-term study. The discussion then shifts to the public and private spheres, without explaining how Kundera's novel is perceived within these two contexts or what the author's view on this is. 

 The article also includes elements that need to be reformulated and re-discussed according to scientific standards. What does “new possible communities” mean, and what is meant by “we need to consider the spatial and cartographic manoeuvres undertaken in this particular genre”? We cannot follow a logical analytical process. “It is easy to foresee that these manoeuvres would also bring the political novel into a triple or threefold relationship with political practises,” the author writes, which reflects a confusion of ideas. 

 The author does not sufficiently clarify how the concept of “deteritorialization”(Guattari and Deleuze) is explained and applied, especially considering that it pertains to an escape from “organized/striated space.” Similarly, the phrase “counter-cartographic” is used but not explained, especially since it does not apply to the chosen novel. 

 We cannot reconcile statements from narratology such as the definition of narrator as “an abstract dimension of the text” with the one related to presence through “his bodily gestures, comments on the timbre of his voice, and behaving like a narrator from the oral tradition of literature.” In the selected novels, as much of literary criticism has pointed out, we are dealing with a clearly abstract narrator, which cannot be negotiated. 

 Such statements as “cartographic hegemonic obsession revealed during the Algerian War, when maps were used as tools of the dominating colonial power” should be developed further, as they fall within the arguments that the author initially announced they would explain. 

 The conclusion does not clarify the argument “To achieve this goal, they need to change our usual way of dealing with literary texts,” as the author does not explain how this change occurs or how this article proposes an alternative method of analyzing the novel.

***My recommendation is that the article should be revised extensively before being indexed. The primary focus of these revisions should be on achieving greater conceptual clarity. Currently, the article introduces some theoretical concepts and references, but these are often presented without clear definitions or precise explanations of how they relate to the overall argument. Clarifying these ideas and selecting appropriate analytical tools will strengthen the foundation of the research, making it easier for readers to follow and appreciate the critical contribution.***

***Once the conceptual framework is clarified, the next step should involve carefully applying these refined concepts to the two selected novels. The current treatment of the novels appears somewhat superficial and fragmented, lacking a coherent connection between theory and literary analysis. By clearly demonstrating how the concepts function in relation to these specific texts, the article would provide a more substantive and nuanced interpretation. This application should serve to illustrate and test the validity of the theoretical framework, ensuring that the analysis remains focused and relevant.***

***Furthermore, the conclusions drawn from this application should be both pertinent and insightful, avoiding redundancy or superficial remarks. They should synthesize the findings from the textual analysis and relate them back to the initial theoretical premises. Well-constructed conclusions will also help highlight the original contributions of the paper, showcasing new perspectives or understandings about the novels under consideration and their relation to broader literary or cultural themes.***

***In summary, the article’s indexing should be contingent on a thorough revision process that emphasizes conceptual precision, careful application of theory to the texts, and well-founded, meaningful conclusions. Such a structured approach will enhance the scholarly value of the work, ensuring it makes a clear, coherent, and original contribution to the field. This will also make the article more accessible and useful to other researchers interested in literary studies and the specific themes addressed.***

If any, are all the source data and materials underlying the results available?

Yes

Is the work original in terms of material and argument?

Yes

Is the work clearly and cogently presented?

Yes

Is the argument persuasive and supported by evidence?

Partly

Does the research article contribute to the cultural, historical, social understanding of the field?

Yes

Does it sufficiently engage with relevant methodologies and secondary literature on the topic?

Partly

Reviewer Expertise:

World Literature Studies, Cultural Studies, Geocriticism, Literary Studies

I confirm that I have read this submission and believe that I have an appropriate level of expertise to confirm that it is of an acceptable scientific standard, however I have significant reservations, as outlined above.

# Reviewer report: Reviewer response for version 1

**DOI:** 10.21956/openreseurope.21552.r54623

**Published:** 2025-05-30

**Authors:** Dawit Dibekulu

**Affiliations:** 1 Bahir Dar University, Bahir Dar, Ethiopia

**Clarity and Presentation**

 The manuscript is lucid, well-organized, and demonstrates a clear line of argumentation. It moves coherently from theoretical framing to textual analysis, maintaining an academic tone that remains accessible to an interdisciplinary readership. The prose is elegant yet precise, and the structure allows readers to follow the author’s conceptual progression with ease.

**Impact and Novelty**

 The article offers a compelling and innovative contribution to literary and political theory. Its central proposition—a threefold interpretive model for political novels—presents a valuable analytical tool:

1. Reproduction of hegemonic perceptions,
2. Critical exposure of ideological manipulations, and
3. Emancipatory cartographies.

Especially noteworthy is the concept of **"cartographies of time,"** which opens fresh dialogue between literary studies, temporality, and spatial-political theory. This synthesis is both original and intellectually generative.

**Methodological Rigor**

 The manuscript demonstrates commendable methodological depth. Theoretical grounding in postcritical literary theory (Rita Felski, Stephen Best), temporal and spatial studies (Gilles Deleuze, Alain Badiou, Rosenberg & Grafton) is robust. The close readings of Kundera and Ernaux are richly textured and persuasive, successfully marrying theory with textual detail.

**Alignment of Conclusions**

 The conclusions flow logically from the analysis. However, the third category— **"emancipatory cartographies"**—would benefit from greater empirical or historical grounding. Presenting at least one concrete example of how a political novel has influenced democratic movements or civic discourse would enhance the argument’s applied dimension.

**2. Detailed Section-by-Section Review**

**Title**

- **Strengths**: The title is clear, compelling, and rich in thematic keywords.
- **Suggestion**: For searchability and precision, consider adding the names *Kundera* and *Ernaux* to the title.

**Abstract**

- **Strengths**: The abstract succinctly outlines the article’s objectives, method, and significance.
- **Suggestion**: Define “cartographies of time” more clearly for non-specialists to ensure accessibility across disciplines.

**Introduction**

- **Strengths**: Strong contextualization of gaps in existing scholarship, especially regarding political temporality in fiction.
- **Suggestion**: A brief acknowledgment of critiques of postcritique (e.g., Marxist or ideological theory-based concerns) would provide a more balanced theoretical positioning.

**Methods**

- **Strengths**: Methodology is appropriate for a humanities article—blending theoretical analysis with interpretive close reading.
- **Suggestion**: Explicitly justify the choice of Kundera and Ernaux. Framing them as exemplars of dissident and feminist writing would enhance methodological transparency.

**Results and Discussion**

- **Strengths**:
  - The reading of Kundera’s *“fur cap”* episode is especially evocative and analytically rich.
  - Ernaux’s fragmented temporality and affective memory politics are interpreted with sensitivity and originality.
- **Critiques**:
  1. The transition between the Kundera and Ernaux sections is abrupt. A bridging paragraph highlighting a shared theme (e.g., materiality, affect, or resistance) would help unify the case studies.
  2. The emancipatory dimension remains somewhat abstract. The author could strengthen this section by referencing a real-world impact (e.g., Kundera’s role in Czech dissent or Ernaux’s influence on feminist memory politics).
  3. The notion of "cartographies of time" would benefit from engaging more directly with recent work on temporality in literature, such as Mark Currie’s *About Time* or Elizabeth Freeman’s *Chrononormativity*.

**Tables, Figures, and Primary Material**

- **Comment**: Although the article lacks visual data, the textual excerpts are well-integrated and support the argument effectively.

**References**

- **Strengths**: The bibliography is comprehensive and includes relevant, recent works.
- **Suggestion**: For balance, include references that critically examine postcritical approaches (e.g., *The Limits of Critique* by Felski or essays from its symposium responses).

**3. Final Recommendations**

**Major Revisions**

1. Expand on the “emancipatory cartographies” with a case study or historical example demonstrating how literary temporalities can incite democratic engagement.
2. Deepen engagement with contemporary temporal theory (e.g., Currie, Freeman) to solidify the novelty of “cartographies of time.”
3. Briefly respond to critiques of postcritique to pre-empt objections and enrich the theoretical discourse.

**Minor Revisions**

1. Smooth the Kundera–Ernaux transition with a thematic link sentence.
2. Clarify the term “cartographies of time” in both the abstract and introduction for broader readability.
3. Consider adjusting the title for keyword optimization and discoverability.

**Strengths to Retain**

- The three-part framework is conceptually robust and offers a new lens for literary political analysis.
- The close readings are meticulous, theoretically sophisticated, and well-matched to the central argument.
- The interdisciplinary engagement is commendable and could stimulate cross-domain academic dialogue.

**4. Overall Assessment**

 This manuscript is a rigorous and thought-provoking contribution to the fields of literary theory, politics, and temporal studies. Its innovative framework and elegant textual analysis ensure it will resonate with scholars interested in literature’s relationship to power, perception, and temporality. With revisions to clarify and support its most ambitious claims, the article is well-suited for *Open Research Europe*

If any, are all the source data and materials underlying the results available?

Yes

Is the work original in terms of material and argument?

Partly

Is the work clearly and cogently presented?

Yes

Is the argument persuasive and supported by evidence?

Yes

Does the research article contribute to the cultural, historical, social understanding of the field?

Partly

Does it sufficiently engage with relevant methodologies and secondary literature on the topic?

Partly

Reviewer Expertise:

Dawit Dibekulu Alem is an Assistant Professor at Bahir Dar University, Faculty of Humanities, Department of English Language and Literature. He was born in Ethiopia and completed his BA in English Language and Literature at Woldia University. He accomplished his MA in TEFL at Debre Markos University in Debre Markos, Ethiopia, and holds a PhD from Bahir Dar University. Dawit is a member of the Reviewer Board for several international journals, including the International Journal of Psychology and Counselling, International Journal of Learning, Teaching, and Educational Research, African Educational Research Journal, Journal of Engineering Research and Reports, International Research Journals, International Journal of English and Literature, and the ACTA Scientific Computer Sciences Review Board. Additionally, he serves on the Editorial Board of PLOS ONE. His research interests include language assessment, literature, language-learning strategies, CALL, material development, psychology, educational psychology, curriculum and syllabus design, social sciences, and other language-related areas. Dawit has published articles in various domestic and international journals and presented papers at numerous conferences.

I confirm that I have read this submission and believe that I have an appropriate level of expertise to confirm that it is of an acceptable scientific standard.
